# Estandarización de zimosán opsonizado como estímulo en la técnica de la 1,2,3-dihidrorrodamina para evaluar el estallido respiratorio del neutrófilo

**DOI:** 10.7705/biomedica.7461

**Published:** 2024-12-23

**Authors:** Uriel Pérez-Blanco, Jenniffer Yissel Girón, Guillermo Juárez-Vega, María Jiménez, Carlos Sánchez, Ricardo Rioja, Sara Espinosa-Padilla, Lizbeth Blancas-Galicia

**Affiliations:** 1 Laboratorio de Inmunodeficiencias, Instituto Nacional de Pediatría, Ciudad de México, México Instituto Nacional de Pediatría Laboratorio de Inmunodeficiencias Instituto Nacional de Pediatría Ciudad de México México; 2 Red de Apoyo a la Investigación, Coordinación de la Investigación Científica, Instituto Nacional de Ciencias Médicas y Nutrición Salvador Zubirán, Universidad Nacional Autónoma de México, Ciudad de México, México Universidad Nacional Autónoma de México Red de Apoyo a la Investigación, Coordinación de la Investigación Científica Instituto Nacional de Ciencias Médicas y Nutrición Salvador Zubirán Universidad Nacional Autónoma de México Ciudad de México México

**Keywords:** enfermedad granulomatosa crónica, rodamina 1,2,3, zimosán, NADPH oxidasa., Granulomatous disease, chronic, rhodamine 1,2,3, zymosan, NADPH oxidase.

## Abstract

**Introduction.:**

Chronic granulomatous disease is a defect in phagocytosis due to deficiency of gp91^*phox*^ , p22^*phox*^ , p47^*phox*^ , p40^*phox*^ , and p67^*phox*^ (classic form of the disease). Recently, EROS and p40phox deficiency were described as responsible for the non-classical form of the disease. The 1,2,3-dihydrorhodamine oxidation technique, with phorbol-12-myristate 13-acetate as a stimulus, is performed to diagnose the classic chronic granulomatous disease. However, oxidation mediated by EROS and p40^*phox*^ requires stimuli such as zymosan, *Escherichia coli*, or *Staphylococcus aureus*.

**Objetivo.:**

Estandarizar el estímulo con zimosán en la técnica de la 1,2,3-dihidrorrodamina para evaluar el estallido respiratorio del neutrófilo.

**Materiales y métodos.:**

Se obtuvo sangre de cinco sujetos sanos previo consentimiento informado. Se utilizó la técnica de 1,2,3-dihidrorrodamina usando el forbol-12-miristato- 13-acetato como control y diferentes cantidades de zimosán opsonizado (150,100, 50, 20 y 10 µg). Por citometría de flujo, se obtuvo la intensidad de fluorescencia media de la rodamina 1,2,3 en las poblaciones de neutrófilos y se calculó el índice de oxidación. Se utilizaron las pruebas de Kolmogorov-Smirnov, ANOVA y el análisis post-hocóe Tukey. Se consideró un valor de p < 0,05 como estadísticamente significativo.

**Resultados.:**

El forbol-12-miristato-13-acetato incrementó la intensidad de la fluorescencia media de la rodamina 1,2,3 en los sujetos sanos. Entre las diferentes condiciones de zimosán probadas, la de 50 µg fue la cantidad óptima y reproducible en todos los controles por el análisis estadístico y los hallazgos citométricos.

**Conclusiones.:**

Se presenta la optimización de la técnica 1,2,3-dihidrorrodamina empleando el zimosán como estímulo. Se propone su implementación en los laboratorios de diagnóstico clínico como parte de la evaluación diagnóstica rutinaria de la enfermedad granulomatosa crónica.

## Abstract (en)

**Objective.:**

To optimize the 1,2,3-dihydrorhodamine technique using zymosan to assess neutrophil respiratory burst and detect the non-classical chronic granulomatous disease.

**Materials and methods.:**

Blood was obtained from five healthy subjects after the signature of the informed consent. The 1,2,3-dihydrorhodamine technique was performed with phorbol-12-myristate-13-acetate as control and different quantities of opsonized zymosan (150, 100, 50, 20, and 10 µg). We obtained through flow cytometry the mean fluorescence intensity of rhodamine 1,2,3 oxidated in the neutrophil population and calculated the oxidation index. The Kolmogorov-Smirnov test, ANOVA, and Tukey’s post-hoc analysis were used. We considered a p value < 0.05 as statistically significant.

**Results.:**

The phorbol-12-myristate-13-acetate increased the rhodamine 1,2,3 mean fluorescence intensity in healthy subjects. Among the different zymosan conditions tested, we selected 50 µg as the optimal and reproducible amount in all controls according to the statistical analysis and cytometric findings.

**Conclusions.:**

We present the optimization of the 1,2,3-dihydrorhodamine technique using zymosan. We propose its implementation in clinical diagnostic laboratories to expand the diagnosis of chronic granulomatous disease.

 La enfermedad granulomatosa crónica es un error innato de la inmunidad caracterizado por la incapacidad de los fagocitos para producir especies reactivas de oxígeno (ROS), debido a una falla del complejo enzimático de la oxidasa del fosfato dinucleótido de la nicotinamida adenina fosfato (NADPH). Este defecto genético conduce a fallas en la expresión de cualquiera de las cinco subunidades que constituyen dicha oxidasa: las transmembrana gp91^*phox*^ y p22^*phox*^ , que forman el flavocitocromo b_558_, y las citosólicas p47^*phox*^ , p40^*phox*^ y p67^phox^. ^1^

En el 2017, se describió otra nueva deficiencia relacionada con otro componente del complejo enzimático NADPH oxidasa: la proteína chaperona EROS (*Essencial for Reactive Oxygen Species*), implicada en la regulación directa de la expresión de gp91^*phox*^ y p22^*phox* (^^2^. Los genes involucrados en las diversas variantes patógenas de la enfermedad granulomatosa crónica son *CYBB, CYBA, NCF1, NCF2, NCF4 y CYBC1*, que codifican para las proteínas gp91^*phox*^ p22^*phox*^ , p47^*phox*^ , p67^*phox*^ , p40^*phox*^ y EROS, respectivamente ^2^^,^^3^.

En el 2019, Dinauer acuñó el término de enfermedad granulomatosa crónica clásica y no clásica ^4^. La forma clásica se caracteriza por una susceptibilidad anormal a infecciones recurrentes, graves e invasivas, causadas por bacterias y hongos, además de hiperinflamación sistémica. Es ocasionada por deficiencia de gp91^*phox*^ , p22^*phox*^ , p47^*phox*^ y p67^*phox* (^^1^^,^^3^. Las manifestaciones infecciosas predominan sobre las inflamatorias en esta forma clásica.

Por otro lado, la enfermedad granulomatosa crónica no clásica, es causada por deficiencia de p40^*phox*^ y EROS ^4^. La deficiencia de p40^*phox*^ se caracteriza por el predominio de los fenómenos inflamatorios en comparación con los infecciosos, como enfermedad inflamatoria intestinal, trombocitopenia autoinmunitaria, lupus discoide, eritema nodoso, úlceras orales y fotosensibilidad ^5^^-^^7^. En la deficiencia de EROS, también predominan las manifestaciones inflamatorias ^8^. Entre las manifestaciones infecciosas, se han descrito susceptibilidad a virus (herpes simple y varicela zóster), infección por *Clostridioides difficile* e infección invasiva por *Streptococcus pneumoniae*, las cuales no se ven en la forma clásica, ni en la deficiencia de p40^*phox* (^^8^^,^^9^. A la fecha, se han reportado 28 casos de deficiencia de p40^*phox (*^^5^^-^^7^^,^^10^^,^^11^ y 13 casos de deficiencia de EROS (^8^^,^^9^^,^^12^^-^^14^. En los cuadros suplementarios 1 y 2 se resumen sus características.

El fundamento de las pruebas diagnósticas para la enfermedad granulomatosa crónica es la medición de la capacidad oxidativa de la NADPH oxidasa en los neutrófilos, mediante la cuantificación del superóxido o el peróxido de hidrógeno (H_2_O_2_). Una de las pruebas diagnósticas es la técnica de oxidación de la 1,2,3-dihidrorrodamina ^3^^,^^15^. La 1,2,3-dihidrorrodamina atraviesa las membranas citoplasmática y vesicular, en donde es oxidada por el H_2_O_2_ a rodamina 1,2,3 en los neutrófilos estimulados ^15^. Cuando la rodamina 1,2,3 es excitada por una longitud de onda de 488 nm, emite una señal fluorescente a 585 nm que puede ser detectada por citometría de flujo ^16^.

Existen diversos estímulos para la liberación de superóxido en las pruebas *in vitro*, entre las cuales se encuentran: zimosán, *Escherichia coli, Staphylococcus aureus* y forbol-12-miristato-13-acetato (PMA) ^6^. Cada uno activa la enzima NADPH oxidasa por diferentes vías: i) E. coli mediante el receptor de tipo toll4 (TLR4) ^17^; ii) S. aureus mediante el TLR1, TLR6, TLR8 y TLR9 ^17^; iii) zimosán mediante TLR2 y TLR6, fosforilando a la subunidad p47^*phox* (^^18^; y iv) PMA que atraviesa la membrana plasmática y activa la proteína cinasa C que, a su vez, fosforila las subunidades de la NADPH oxidasa ^19^.

Las formas clásicas de la enfermedad granulomatosa crónica pueden detectarse con la técnica de 1,2,3-dihidrorrodamina usando zimosán, *E. coli, S. aureus* y PMA ^15^. Las formas no clásicas, recientemente descritas, pueden detectarse con la misma técnica y estímulos, excepto el PMA (figura 1) ^6^^-^^9^. En los laboratorios de diagnóstico de diferentes partes del mundo, en dicha técnica se utiliza únicamente PMA como estímulo, por lo que el diagnóstico de las formas no clásicas se pasa por alto ^15^. En la literatura consultada, no está descrita la técnica de oxidación de 1,2,3-dihidrorrodamina usando zimosán como estímulo adicional, para su uso en los laboratorios de diagnóstico.

**Figura 1.**
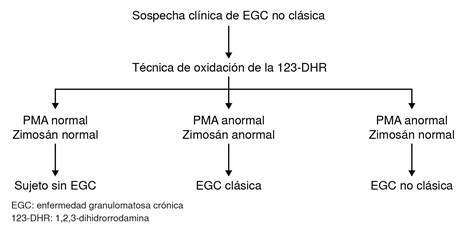
Flujograma del diagnóstico de la enfermedad granulomatosa crónica con la técnica de 1,2,3-dihidrorrodamina, usando dos estímulos: forbol-12-acetato-13-miristato (PMA) y zimosán. En un sujeto sano hay producción de especies reactivas de oxígeno con PMA y zimosán; en un paciente con enfermedad granulomatosa crónica clásica no hay producción de especies reactivas de oxígeno con los dos estímulos, y en un paciente con la forma no clásica de la enfermedad, la producción de especies reactivas de oxígeno es normal con zimosán, pero nula con PMA. EGC: enfermedadgranulomatosa crónica 123-DHR: 1,2,3-dihidrorrodamina

En el presente estudio se sugiere que, en los laboratorios de diagnóstico, para esta técnica no se use solo el PMA como estímulo principal, sino también, el zimosán. En este artículo, se describe la optimización de la técnica de la oxidación de 1,2,3-dihidrorrodamina usando zimosán opsonizado.

## Materiales y métodos

### Recolección de sangre periférica

Para seleccionar los sujetos sanos, una médica especialista en inmunología elaboró la historia clínica y realizó el examen físico de los candidatos. Los sujetos seleccionados como sanos tenían un índice de masa corporal dentro de los rangos normales, no eran fumadores, no tomaron medicamentos en el último año y no padecían enfermedades de ningún tipo.

La sangre periférica de cinco adultos jóvenes aparentemente sanos, dos hombres y tres mujeres de origen mexicano, se recolectó en tubos con heparina de litio o de sodio. Las muestras se obtuvieron por venopunción periférica y fueron procesadas inmediatamente. Previamente, se firmó el consentimiento informado. Este proyecto fue aceptado por los comités de ética, investigación y bioseguridad del Instituto Nacional de Pediatría: INP- 030/2020.

### Reactivos

Las concentraciones, diluciones, alícuotas y métodos de conservación usados en la preparación de los reactivos, como dihidrorrodamina, forbol-12- miristato-13-acetato, zimosán opsonizado, tampón de fosfato salino (PBS IX), solución de lisis y paraformaldehído, se describen con detalle en el cuadro 1.

**Cuadro 1.** Lista y descripción de los reactivos

Reactivos	Referencia, marca y país de fabricación	Concentración/ presentación	Preparación	Conservación
1,2,3-dihidrorrodamina	D23806 Thermo Fisher Scientific® USA	5 mM estabilizado en 1 mi de dimetilsulfóxido	Realizar alícuotas de 2 µl*. Al momento de usarlo, agregar 38 µl de PBS, mezclar y homogeneizar	-20 °C
Forbol-12-miristato-13 acetato (PMA)	V1171 Promega® USA	5 mg liofilizado. Solución madre preparada a una concentración de 5 mM	Agregar al liofilizado 1,62 ml de DMSO. Realizar alícuotas de 2 µl. Al momento de usarlo, agregar 198 µl de PBS, mezclar y homogeneizar.	-30 a -10 °C
Zimosán A	Z4250 Sigma Aldrich® USA	250 mg	Preparación de zimosán opsonizado.	2 a 8 °C
			Por cada 1 mg de zimosán agregar 100 µl de suero humano en un microtubo, homogeneizar e incubar a 37ºC por una hora.	
			Nota: el suero humano de un sujeto sano fue congelado en alícuotas de 100 µl. Se descongeló al momento de ser requerido.	
Tampón fosfato salino (PBS) 1X^⸿^	BP2438-4	137 mM NaCI		15 a 25°C
	Thermo Fisher Scientific^®^ USA	2.7 mM KCI		
		10 mM Na_2_HP0_4_		
		1.8 mM KH_2_P0_4_		
BD FACS Solución de lisis 10X	349202 BD Biosciences^®^ USA	100 ml	Preparar a 1X	2 a 25 °C
Paraformaldehído (4 %)	P6148 Sigma Aldrich^®^ USA	100 g	4 g de polvo en 100 ml de PBS	25 °C

DMSO: dimetilsulfóxido; PBS: tampón fosfato salino; NaCI: cloruro de sodio; KCI: cloruro de potasio; Na_2_HP0_4_: fosfato sódico; KH_2_P0_4_: fosfato monopotásico

* Para evitar la degradación de la 1,2,3-dihidrorrodamina por ciclos de congelación y descongelación ^11^ Para evitar la degradación del PMA por ciclos de congelación y descongelación

^⸿^ Se puede reemplazar por solución con concentración del 0,9 % de NaCI

**Figura 2.**
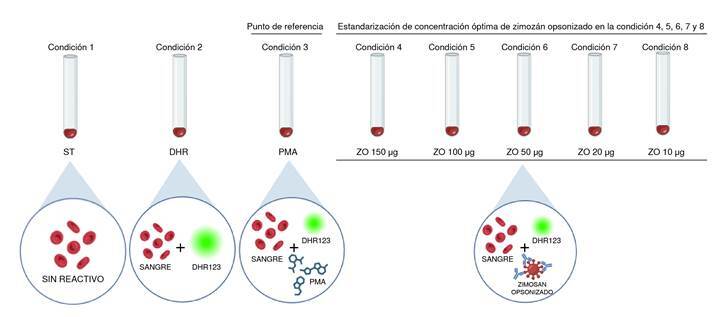
Esquema de trabajo de la optimización de la prueba de 1,2,3-dihidrorrodamina con zimosán opsonizado. Las cantidades decrecientes de zimosán se muestran a partir de la condición 4. La condición 3 (PMA) fue el estímulo de referencia. ST: sintinción; PMA: forbol-12-acetato-13-miristato; ZO: zimosán opsonizado; 1,2,3-DHR: 1,2,3-dihidrorrodamina.

*Optimización de la técnica de oxidación de la 1,2,3-dihidrorrodamina con zimosán en sangre periférica*. Para la citometría de flujo, cada muestra sanguínea requirió ocho tubos, los cuales fueron rotulados según la condición (C) del experimento de Cl a C8 (figura 2). A cada tubo se le agregaron 50 µl de sangre y, luego, a los tubos C2 a C8, 5 µl de 1,2,3-dihidrorrodamina. Se evitó exponerlos a la luz hasta finalizar la técnica, debido al fenómeno de desvanecimiento de la fluorescencia de un fluorocromo por exposición a la luz (*quenching*).

Los tubos se incubaron a 37 ºC durante cinco minutos y después se agregaron los estímulos (su preparación se describe en el cuadro 1). A los tubos C3, se les adicionaron 30 µl de la preparación de PMA. A los otros, se les agregaron diferentes cantidades de zimosán opsonizado con el objetivo de elegir la condición óptima, así: C4, 150 µg (15 µl); C5, 100 µg (10 µl); C6, 50 µg (5 µl); C7, 20 µg (2 µl), y C8, 10 µg (1 µl).

Todos los tubos (C1 a C8) se incubaron a 37 ^S^C durante 30 minutos; al finalizar la incubación, se agregaron 1.000 µl de solución de lisis de eritrocitos IX, se homogenizó la mezcla y se incubó durante 10 minutos entre 2 y 4 ºC, cubierta con papel aluminio. Tras la incubación, se centrifugaron todos los tubos a 1.500 rpm a 21 °C durante cinco minutos. Después de la centrifugación, todos los tubos se sometieron a decantación y lavado con 1.000 µl de solución tampón PBS IX. Se repitieron la centrifugación y la decantación. Las células contenidas en los tubos se fijaron con 150 µl de 4 % de paraformaldehído.

*Citometría de flujo*. Se utilizó un citómetro de flujo LSR Fortessa™ (BD Biosciences^®^) de cuatro láseres. Se adquirieron 10.000 eventos de la región de neutrófilos seleccionada mediante el *software* FACSDiva™ (BD Biosciences^®^). El análisis de los resultados obtenidos en formato FCS (*Flow Cytometry Standard*) se hizo con el programa FlowJo™, versión 8.7, para Mac iOS. Se obtuvo el promedio de la intensidad de fluorescencia media (IFM). Para calcular el índice de oxidación, se dividió el promedio de la intensidad de fluorescencia media de los tubos con estímulo (C3 a C8), por la del tubo sin estímulo (C2).

### Análisis estadístico

Se utilizó la prueba estadística de Kolmogorov-Smirnov para conocer la distribución de las variables. Las fluorescencias medias obtenidas mediante diferentes cantidades de zimosán, se compararon mediante un ANOVA de una vía con análisis post-hoc de Tukey. Se consideró el valor de p < 0,05 como estadísticamente significativo. Los datos se analizaron en los programas SPSS™, versión 25 (SPSS Inc., Chicago, IL) y GraphPad Prism™, versión 9.0 (GraphPad Sofware, La Jolla California, USA).

### Consideraciones éticas

Los autores declaran que los procedimientos se llevaron a cabo con apego a las normas éticas, el Reglamento de la Ley General de Salud en materia de Investigación para la Salud, y la Declaración de Helsinki. El estudio fue aprobado por el Comité de Investigación del Instituto Nacional de Pediatría (INP-030/2020). Los autores declaran que han seguido los protocolos de su centro de trabajo sobre la publicación de datos de pacientes.

## Resultados

La producción de especies reactivas de oxígeno mediada por PMA en la técnica de la oxidación de la 1,2,3-dihidrorrodamina se ha validado en múltiples artículos de investigación ^5^^,^^6^, de tal forma que la intensidad de fluorescencia media producida por el PMA en este ensayo fue utilizada como punto de referencia. La cantidad de 0,3 ng/pl de PMA generó un incremento en la fluorescencia debido a la oxidación de la 1,2,3-dihidrorrodamina a rodamina 1,2,3 en todos los sujetos sanos (IFM = 9.691). Lo anterior sirvió para validar este estímulo como referencia para la optimización de la prueba, pero con zimosán opsonizado (figura 1 suplementaria).

*Reacción oxidativa con diferentes diluciones seriadas de zimosán opsonizado.* En los cinco sujetos sanos, las diferentes concentraciones de zimosán, produjeron las siguientes medias de intensidad de fluorescencia de rodamina 1,2,3: 150 µg, 3.946 ± 537,7; 100 µg, 4.046 ± 459,7; 50 µg, 3.923 ± 941,4; 20 µg, 3.092 ± 673,7; y 10 µg, 1.086 ± 241,7.

Al comparar las medias de la intensidad de fluorescencia de las concentraciones de 150,100, 50, 20 y 10 gg, se halló que eran estadísticamente significativas, con un valor de p = 0,0012. Por lo tanto, se procedió a realizar un análisis post-hoc entre grupos. No se observaron diferencias significativas al comparar las medias de intensidad de fluorescencia entre 150 y 100,100 y 50, y 50 y 20 µg.

**Figura 3.**
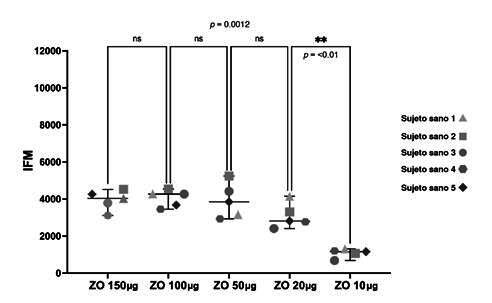
Representación gráfica de la distribución de la intensidad de fluorescencia media de rodamina 1,2,3 como respuesta a diferentes cantidades de zimosán opsonizado. Se observó una disminución gradual de la intensidad de fluorescencia media directamente proporcional a la cantidad de zimosán. El valor de p compara la media de intensidad de fluorescencia de todas las cantidades de zimosán (10, 20, 50, 100 y 150 µg). Entre las cantidades de 10 y 20 µg hay una diferencia estadísticamente significativa (p < 0,01). La línea vertical entre las figuras geométricas representa la desviación estándar y, la línea horizontal, la intensidad de fluorescencia media.

**Figura 4.**
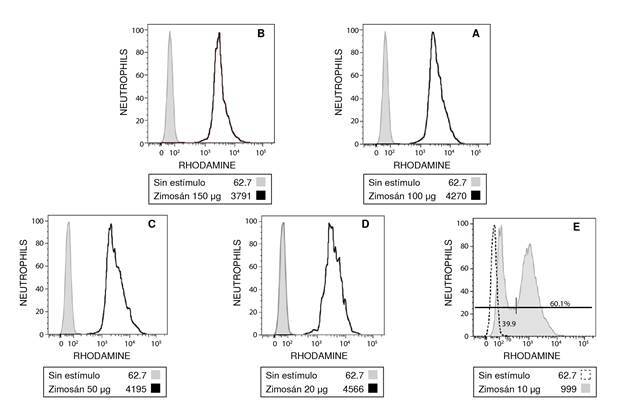
Histogramas que muestran neutrófilos estimulados con diferentes cantidades de zimosán opsonizado. La línea gris muestra la condición sin estímulo y la línea negra ilustra la condición con zimosán opsonizado. En el recuadro inferior de cada histograma se muestran las cantidades de zimosán opsonizado: A) 150 µg, B) 100 µg, C) 50 µg, D) 20 µg y E) 10 µg; y la intensidad de fluorescencia media de la rodamina 123. En la figura E, la cantidad de 10 µg no fue suficiente para activar la producción de especies reactivas de oxígeno en el 100 % de los neutrófilos, por lo que se observó un patrón bimodal de rodamina 123. IFM: intensidad de fluorescencia media; ZO: Zimozan opsonizado; ns: no significativo ** p< 0,01;

Por otro lado, al comparar 20 y 10 µg, sí se encontraron diferencias estadísticamente significativas (p ¿ 0,01) (figura 3). El análisis por citometría de flujo mostró que la cantidad de 10 µg no fue suficiente para activar a todos los neutrófilos, el 40 % no se activó y el 60 % sí (figura 4).

### Índices de oxidación con PMA y zimosán opsonizado

Al comparar los promedios de los índices de oxidación (división del promedio de la intensidad de fluorescencia de la condición con estímulo por la intensidad de fluorescencia media del tubo sin estímulo) obtenidos con las cantidades de zimosán, se seleccionó la cantidad de 50 µg porque, a partir de 20 µg, la intensidad de la fluorescencia media empieza a disminuir y la desviación estándar es más amplia (cuadro 2).

**Cuadro 2:** índices de oxidación con PMA y zimosán opsonizado

Estímulo	Cantidad	S1	S2	S3	S4	S5	Media	DE
PMA	0,3 ng/µl	185	116	144	189	135	154	-
Zimosán opsonizado	150 µg	83	45	60	61	64	62,6	13,58
	100 µg	89	45	68	68	55	65	16,54
	50 µg	65	52	70	57	58	60,4	7,09
	20 µg	86	33	38	54	42	50,6	21,26
	10 µg	2	10	10	23	17	17,4	7,63

**Figura 5.**
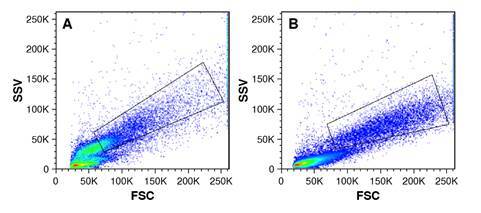
Dispersión de neutrófilos en función del tamaño y la granularidad. En las gráficas de puntos, se observan diferentes patrones de dispersión entre la respuesta a PMA (A) y a 50 µg de zimosán opsonizado (B). Los neutrófilos se muestran dentro del recuadro negro FSC: forwardscatter, SSC: side scatter, PMA: forbol-12-miristato-13-acetato

S: sujeto sano; DE: desviación estándar; PMA: forbol-12-acetato-13-miristato;

*Patrón de tamaño contra granularidad entre PMA y zimosán opsonizado*. Se observó que los neutrófilos tienen un patrón de dispersión celular (tamaño contra granularidad) diferente entre la condición activada con PMA y aquella con zimosán opsonizado (figura 5).

## Discusión

Recientemente, se han descrito las deficiencias de EROS y p40^*phox*^ como causas genéticas de la enfermedad granulomatosa crónica no clásica ^4^^,^^8^. En las descripciones iniciales, se realizaron diversos experimentos para corroborar la correlación entre fenotipo y genotipo y una de ellas fue la medición de especies reactivas de oxígeno mediante la prueba de oxidación de la 1,2,3- dihidrorrodamina ^4^^,^^8^.

En los pacientes con la forma no clásica de la enfermedad, la producción de especies reactivas de oxígeno es normal usando PMA como estímulo en la prueba, pero nula con zimosán, *E colio S. aureus*^4^. La prueba de 1.2.3- dihidrorrodamina en los laboratorios clínicos se hace con PMA, con lo cual, el diagnóstico de las deficiencias de EROS y p40^*phox*^ se pasa por alto.

La propuesta de este estudio es realizar la técnica de oxidación de la 1.2.3- dihidrorrodamina tanto con PMA como con un segundo estímulo, ya sea zimosán, *E. eolio S. aureus*. Aquí se presenta una optimización de la técnica de oxidación de la 1,2,3-dihidrorrodamina con zimosán opsonizado que puede ser de utilidad para identificar las formas no clásicas de la enfermedad granulomatosa crónica (cuadro 3). Se seleccionó el zimosán porque es más accesible en el mercado y de menor costo que *E. coli* y; además, tiene menos riesgos biológicos para el laboratorio clínico ^20^^,^^21^. Es importante resaltar que el zimosán debe ser opsonizado con suero humano para estimular la producción de especies reactivas de oxígeno.

**Cuadro 3:** Pasos de la técnica de oxidación de la 1,2,3-dihidrorodamina en la estandarización final

1	Opsonizar el zimosán con suero humano según el cuadro 1.
	Incubarlo durante una hora a 37 °C.
	Mientras se incuba, realizar los pasos 2 a 3.
2	Preparar la 1,2,3-dihidrorrodamina según cuadro 1.
	Agregar 50 µl de sangre humana a tubos rotulados como condiciones C1, C2, C3 y C4.
	Añadir 5 µl de preparación de 1,2,3-dihidrorrodamina a los tubos de las condiciones C2, C3, y C4, mezclar e incubar durante cinco minutos a 37 °C.
3	Preparar el primer estímulo: PMA según el cuadro 1.
4	Al término del paso 2, agregar 30 µl de preparación de PMA a un tubo rotulado como C3 y mezclar.
	Añadir 5 µl (50 µg) de zimosán opsonizado al tubo rotulado como C4.
	Incubar los tubos C1, C2, C3 y C4 durante 30 minutos a 37 °C.
5	Al término de la incubación, agregar 1 ml de 1X de solución de lisis de eritrocitos a los tubos C1, C2, C3 y C4, mezclar e incubar en refrigeración a 4 °C durante 10 minutos.
6	Centrifugar los tubos C1, C2, C3 y C4 a 1.500 rpm durante cinco minutos.
	Decantar y añadir 1 ml de PBS a cada condición para lavar.
7	Centrifugar de nuevo a 1.500 rpm durante cinco minutos.
	Decantar y fijar con 150 µl de 4 % de paraformaldehído.
8	Adquirir inmediatamente los eventos por citometría de flujo y proceder al análisis de los archivos,

El patrón de dispersión de tamaño contra la granularidad que se observa en la adquisición de los eventos en el citómetro de flujo es diferente entre el zimosán y el PMA. Entre las diferentes concentraciones de zimosán opsonizado analizadas en este estudio, se encontró que la cantidad de 50 µg (tubo C6) era la óptima por el análisis estadístico y la reacción oxidativa. Por lo tanto, se propone que sea utilizada en el laboratorio clínico.

En cuanto a la intensidad de la fluorescencia media, la producida con PMAfue mayor que con zimosán, en consecuencia, los índices de oxidación son menores. En el mercado existe un kit comercial (PHAGOBURST™), (ORPEGEN Pharma, Heidelberg, Alemania) que mide la producción de especies reactivas de oxígeno utilizando *E. coli* como estímulo, por lo que es útil para el diagnóstico de la enfermedad granulomatosa crónica no clásica, sin embargo, su costo es elevado, por lo que resulta inaccesible para las instituciones con recursos limitados ^22^.

Actualmente, existen estudios genéticos innovadores, como la secuenciación de nueva generación (genoma, exorna o paneles de genes) para el diagnóstico de los errores innatos de la inmunidad. Los resultados pueden ser variantes patógenas o inciertas ^23^. Para las variantes de significancia incierta en los genes de *CYBC1* (EROS) y *NCF4* (p40^*phox*^ ), la prueba de la 1,2,3-dihidrorrodamina con zimosán es útil para establecer si es patógena o si no lo es.

Finalmente, los autores de este estudio consideran que la técnica de oxidación de la 1,2,3-dihidrorrodamina con zimosán opsonizado puede ser una herramienta útil para identificar las formas no clásicas de la enfermedad granulomatosa crónica. En este trabajo, se presenta la optimización de la técnica de oxidación de la 1,2,3-dihldrorrodamina con zimosán opsonizado, además del ya establecido con PMA. Se propone su implementación en los laboratorios de diagnóstico clínico para el diagnóstico de la enfermedad granulomatosa crónica no clásica.

## Figures and Tables

**Figura suplementaria 1.**
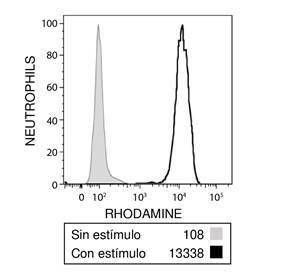
Activación de la NADPH oxidasa como respuesta al forbol-12-miristato-13-acetato (PMA) como estímulo de referencia. EI histograma de color gris representa la intensidad de la fluorescencia media basal de los neutrófilos con 1,2,3-dihidrorrodamina. EI histograma de color negro representa la señal producida por la estimulación con PMA. Se observa un incremento de la intensidad de fluorescencia media en un orden de magnitud de 10^3^.

## Archivos suplementarios

**Cuadro suplementario:** 1. Casos reportados de deficiencia de p40^phox^ en la literatura

Paciente (referencia)	Sexo	Origen	Variante patógena	Consanguinidad o endogamia	Edad de presentación	Edad media de diagnóstico	Espectro clínico	Exámenes paraclínicos y gabinete	TCPH / Seguimiento
P1 ^5^	M	N/D	p.R105Q/p. K52Rfs*79	No	2 años	5 años	Colitis granulomatosa crónica, eccema perioral, úlceras aftosas orales, sinusitis y laringotraqueítis aguda recurrente	Endoscopia: erosiones en fondo gástrico, ulceraciones aftosas difusas en mucosa colónica; biopsia de colon: granulomas epiteliales	No / N/D
P2^6^	M	Pakistán	c.118-1G>A	Sí	4 años	9 años	Úlceras orales, enfermedad inflamatoria intestinal (colitis granulomatosa), abscesos cutáneos por S. aureus.	ANAs (-)	No / Vivo
P3^6^	F	Pakistán	P.R105Q	Sí	9 años	14 años	Lesiones cutáneas de tipo lupus	ANAs (-)	No / Vivo
P4^6^	M	Pakistán	P.R105Q	Sí	Desconocida	46 años	Lesiones cutáneas de tipo lupus en la nfancia	ANAs (-),anti-dsDNA (-)	No / Vivo
P5^6^	M	Pakistán	P-R105Q	Sí	5 años	23 años	Infecciones cutáneas recurrentes por SAMR, lesiones eccematosas	ANAs (-), anti-dsDNA (-)	No / Vivo
P6^6^	M	Pakistán	P-R105Q	Sí	Desconocida	19 años	Enfermedad granulomatosa en encías y piel	BAAR y Ziehl Neelsen (-),ANAs (-)	No / Vivo
P7^6^	M	Pakistán	p.R105Q	Sí	7 años	16 años	Infecciones cutáneas recurrentes, lesiones cutáneas de tipo lupus, dermatitis seborreica	N/D	No / Vivo
P8^6^	F	Pakistán	P.R105Q	Sí	3 años	15 años	Impétigo, lupus discoide	Anti-AMHR (-), anti-Smith (-), anti-reticulina (-), anti- ACPE (-)	No / Vivo
P9^6^	F	Pakistán	P.R105Q	Sí	4 años	9 años	Abscesos cutáneos por SAMR y SGB, impétigo, lesiones cutáneas tipo lupus	ANAs (-), anti-dsDNA (-), anti-ATP (-), anti-Clq (-)	No / Vivo
P10^6^	M	Portugal	c.120_134del	No	5 años	7 años	Abscesos cutáneos y odontogénicos, periodontitis	N/D	No / Vivo
P11^6^	F	Portugal	c.120_134del	No	1 año	1 año	Úlceras orales recurrentes	N/D	No / Vivo
P12^6^	M	Estados Unidos	c.118-1G>A c.759-1 G>C	No	7 años	10 años	Lupus eritematoso cutáneo discoide con sobreinfección por S. aureus, fisuras anales, úlceras orales	Biopsia: criptitis en colon descendente, sigmoides y recto	Sí / Vivo
P13^6^	F	Pakistán	c.118-1G>A	Sí	1 año	2 años	Fiebre crónica, neumonías recurrentes (sin cultivos positivos), granulomatosis de nodulo pulmonar	ANAs (-), ANCA (-), ENAs (-), anti-dsDNA (-). Anti- cardiolipina IgG = 87,6 (0-5,7)	No / Vivo
P14^6^	M	Pakistán	c.118-1G>A	Sí	N/A	1 año	Asintomático	N/A	No / Vivo
P15^6^	F	Pakistán	c.32-2T>G	Sí	2 años	5 años	Infecciones respiratorias recurrentes, bronquiectasias, linfadenitis con abscesos	Lavado broncoalveolar: cepas virales no especificadas y Candida spp.; tomografía computarizada: consolidación pulmonar bilateral densa; biopsia pulmonar: inflamación intersticial crónica. ANAs (+), ANCA (+).	Sí / Vivo
P16^6^	M	Colombia	p.R58C	No	2 años	6 años	Fiebre crónica, diarrea crónica, candidiasis oral (sin resultados microbiológicos), histoplasmosis diseminada recurrente (en ganglios linfáticos, hígado, bazo, orina, plasma y médula ósea)	N/D	No / Vivo
P17^6^	M	Colombia	p.R58C	No	N/A	10 años	Asintomático	N/A	No / Vivo
P18^6^	F	Colombia	p.R58C	No	N/A	8 años	Asintomático	N/A	No / Vivo
P19^6^	M	Colombia	p.R58C	No	N/A	3 años	Asintomático	N/A	No / Vivo
P20^6^	F	Argentina	p.P144T	No	1 año	7 años	Neumonía, linfadenitis regional por BCG, lesiones cutáneas granulomatosas, meningitis por el complejo Mycobacterium avium	N/D	No / Vivo
P21^6^	F	Kuwait	p.W293*	Sí	14 años	37 años	Gastritis, fistula perianal compleja grave con estenosis anal y abscesos perianales recurrentes	Biopsia: adenocarcinoma del canal anal	Sí / Vivo
P22^6^	F	Kuwait	p.W293*	Sí	8 años	32 años	Ulceraciones esofágicas, fístula perianal grave diagnosticada como enfermedad de Crohn, abscesos cutáneos recurrentes, epiescleritis, periodontitis grave	N/D	Sí / Vivo
P23^6^	F	Portugal	c.120_134del	No	2 años	15 años	Enfermedad inflamatoria intestinal con granuloma en colon, liquen plano vulvar	ASCA (+)	No / Vivo
P24^6^	F	Rusia (nacido en Alemania)	c.118-1G>A	No	11 años	17 años	Enfermedad inflamatoria intestinal con granuloma localizado en mucosa oral, esófago y colon, blefaritis y conjuntivitis recurrente	N/D	No / Vivo
P25^6^	F	Chile	p.R58C	No	17 años	23 años	Colitis granulomatosa, enfermedad perianal grave, eritema nudoso y pioderma gangrenoso	N/D	No / Vivo
P26^10^	M	N/D	c.179G>A	N/D	4 años	4 años	Colitis granulomatosa resistente, enfermedad de Crohn, absceso perianal, osteomielitis púbica	Colonoscopia: ulceraciones profundas lineales en colon; biopsia de colón: inflamación transmural grave con granulomas grandes	Sí / Vivo
P27^11^	F	Pakistán	c. 118-1 G>A	Sí	Desconocida	37 años	Neumonía necrotizante por Burkholderia multivorans, tuberculosis no especificada en la infancia	Lavado broncoalveolar: aislamiento de Burkholderia multivorans; cultivo de esputo con P. aeruginosa.	No / Vivo
P28^7^	F	Francia	c.577del c.529-2A>T	No	Desconocida	11 años	Úlceras orales recurrentes, perniosis, BCGitis,	CD4+ naive y CD8+ de memoria elevados,	No / Vivo

N/D: no determinado; N/A: no aplica; SARM: Staphylococcus aureus resistente a meticilina; TCPH: trasplante de células progenitoras hematopoyéticas; ANAs: anticuerpos antinucleares; dsDNA: anticuerpos anti-DNA de doble cadena; BAAR: bacilos ácido-alcohol resistentes; AMHR: anticuerpos antimicrosoma de hígado y riñón; ACPE: anticuerpos anticélulas parietales de estómago; EGB: estreptococo del grupo B; ATP: anticuerpos antitiroperoxidasa; ANCA: anticuerpos anticitoplasma de neutrófilos; ANE: anticuerpos nucleares extraíbles; ASCA: anticuerpos anti-*Saccharomyces cerevisiae*; BCG: bacilo de Calmette-Guérin

**Cuadro suplementario 2.** Casos reportados de deficiencia de EROS en la literatura científica

Paciente (referencia)	Sexo	Origen	Variante patógena	Consanguinidad o endogamia	Edad de presentación	Edad media de diagnóstico	Espectro clínico	Exámenes paraclínicos y gabinete	TCPH / Seguimiento
P1^8^	M	Islandia	p.Tyr2*	N/D	2 años	15 años	Linfadenitis aguda de cara y cuello, neumonía por *Legionella* spp., infecciones de piel y tejidos blandos, enfermedad de Crohn, úlceras orales, abscesos anales, fistula y fisura anal, pancreatitis aguda	Lesiones granulomatosas en biopsia intestinal, aislamiento de *Legionella* spp. y *Burkholderia* spp. de cultivo de esputo	Sí / Vivo
P2^8^	M	Islandia	p.Tyr2*	N/D	6 años	16 años	Otitis media supurativa recurrente, enfermedad de Crohn, abscesos cutáneos, hepatoesplenomegalia grave, infección intestinal por Clostridium difficile, candidemia, pancreatitis aguda	Lesiones granulomatosas en biopsia intestinal	Sí / Fallecido
P3^8^	M	Islandia	p.Tyr2*	N/D	4 años	N/D	Fisuras anales, absceso anal	N/D	No / N/D
P4^8^	F	Islandia	p.Tyr2*	N/D	N/D	23 años	Infección viral por herpes simple, eccema	Factor reumatoide (+)	N/D
P5^8^	M	Islandia	p.Tyr2*	N/D	N/D	N/D	Colitis ulcerativa, diarrea infecciosa, onicomicosis	N/D	N/D
P6^8^	M	Islandia	p.Tyr2*	N/D	N/D	N/D	Enfermedad de Crohn, diarrea infecciosa, tonsilitis crónica, síndrome nefrítico, infección por herpes simple, varicela zóster	N/D	N/D
P7^8^	F	Islandia	p.Tyr2*	N/D	N/D	N/D	Infección invasiva por *Streptococcus pneumoniae*, neumopatía intersticial con fibrosis	S. pneumoniae (+) en hemocultivo	No / Fallecido
P8^8^	F	Islandia	p.Tyr2*	N/D	N/D	N/D	Tuberculosis miliar por *M. tuberculosis*	N/D	N/D
P9^9^	M	ArabiaSaudita	c.127G>A	Sí	2 meses	8 años	BCGitis, neumonías recurrentes, talla baja, faringitis recurrente	Hipogammaglobulinemia, linfopenia, anemiahemolítica utoinmunitaria	Sí /Vivo
P10^12^	M	Islandia	N/D	No	6 años	14 años	Enfermedad de Crohn, neumonía por *Legionella* spp. y *Burkholderia* spp.	Aislamiento de *Legionella* spp. y *Burkholderia* spp. de cultivo de esputo	Sí / Vivo
P11^12^	M	Islandia	N/D	No	11 años	16 años	Otitis media recurrente, enfermedad de Crohn, pancreatitis aguda, hepatoesplenomegalia	N/D	Sí / Fallecido
P12^13^	F	Italia	c.8_7delCTCTCGGGATGTACC	No	2 años	9 años	Fiebre prolongada, hepatoesplenomegalia, enfermedad de Crohn, neumonía basal, aspergilosis	Neopterina, PCR y VSG elevados, calprotectina fecal (+), biopsia intestinal: granulomas epiteliales basales, células gigantes multinucleadas; tomografía computarizada: linfadenopatias difusas; biopsia pulmonar: granulomas epiteliales, células gigantes y necrosis caseosa; *Aspergillus* spp. (+)	No / Vivo
P13^14^	F	Reino Unido	c.327dupp.Val110Cysfs*40	No	5 años	2 años	Sarcoidosis, neumonía por *Malassezia* restricta, hepatitis granulomatosa, sepsis por *Salmonella* spp., infección del sitio quirúrgico por *M. tuberculosis*, osteomielitis, uveítis	*Salmonella* spp. (+) en hemocultivo; biopsia de médula ósea: infiltración granulomatosa; tomografía computarizada: bronquiectasias por tracción, múltiples nódulos linfáticos y parenquimatosos y derrame pleural derecho	No / Vivo

N/D: no determinado; TCPH: trasplante de células progenitoras hematopoyéticas; PCR: proteína C reactiva; VSG: velocidad de sedimentación globular
